# Practical approach to determine apparent digestibility of canine diets*

**DOI:** 10.1017/jns.2014.26

**Published:** 2014-09-25

**Authors:** Esther A. Hagen-Plantinga, Guido Bosch, Wouter H. Hendriks

**Affiliations:** 1 Faculty of Veterinary Medicine, Utrecht University, Yalelaan 7, 3584 CL Utrecht, The Netherlands; 2 Animal Nutrition Group, Wageningen University, PO Box 338, 6700 AH Wageningen, The Netherlands

**Keywords:** Dogs, Apparent digestibility, Titanium oxide, Practical methods, BW, body weight, CF, crude fat, GE, gross energy, TiO_2_, titanium oxide

## Abstract

A practical approach to determine apparent faecal digestibility using privately owned dogs may be a useful tool in evaluating differences in nutrient digestibility between dogs with various life stages. The aim was to develop a simple method that would suit such studies using the whitening agent titanium oxide (TiO_2_) as an indigestible marker. Forty privately owned, healthy male and female dogs of various breeds were included. Selection was based on an owner questionnaire. Means with their standard errors age and body weight (BW) of the dogs were 6·2 (0·6) years (range 1·0–13·0 years) and 22·3 (2·5) kg (range 5·0–43·2 kg), respectively. Owners were provided a commercial dry extruded diet supplemented with a commercially available TiO_2_ containing kibble (final dietary TiO_2_ content: 0·77 g/kg). Dogs were fed the diet for seven consecutive days at 480 kJ × BW^0·75^. On day 7, owners were asked to collect all faeces during 24 h and store faeces at −20°C. Faecal samples were analysed for DM, ash, N, crude fat (CF), crude fibre and Ti and gross energy (GE) and organic matter were calculated. Means with their standard errors apparent faecal digestibility of GE, DM, organic matter, N, CF and crude fibre was 83·7 (0·71), 77·4 (0·79), 83·0 (0·61), 77·7 (0·81), 94·3 (0·51) and 30·3 (4·85), respectively. No significant differences were observed in nutrient digestibility due to weight, age, sex or neuter status. The digestibility assay using a practical approach described here may be a promising tool to determine digestibility of dietary nutrients under free-living conditions. Owner compliance, however, is a potentially limiting factor.

Directed breeding efforts by human subjects since the domestication of dogs led to the dramatic differences in size, weight and activity between today's dog breeds^(^^1^^)^. There are limited data available in the literature on the effect of these differences on nutrient and energy metabolism^(^^2^^,^^3^^)^. Most data in the literature are derived from controlled studies under laboratory conditions, with a limited number of dogs, and with total faecal collection as apparent digestibility method. However, this method is labour-intensive and cannot be used to study breed, age and life stage differences in large groups of privately owned dogs. A reliable digestibility method that can be used under practical conditions may be a useful tool for this purpose. Such a method could provide valuable information on the effect of differences in living conditions (e.g. housing, feed intake, body condition score and activity) on apparent nutrient digestibility. In addition, the use of kennelled laboratory dogs for the purpose of determining apparent nutrient digestibility of diets could be reduced. Ultimately, data derived from the large-scale testing of apparent digestibility under practical living conditions could provide valuable insights for formulation of complete and balanced diets for different dog breeds.

The aim of this study was to develop a simple method that may be used in privately owned dogs, using titanium dioxide (TiO_2_) as an indigestible marker. The effect of body weight (BW), age, sex and neuter status of the privately owned dogs on gross energy (GE) digestibility data derived from this method was researched and compared with current literature using standardised techniques.

## Materials and methods

### Animals

Participation in this study was voluntary and informed consent was obtained from dog-owners who were recruited through national advertisement. Selection of eligible dogs was based on an owner questionnaire. Dogs were excluded from the study if they were younger than 1 year of age, not healthy or receiving drugs. Dogs were considered to be healthy when clinical signs related to systemic disease were absent. Forty healthy, privately owned male and female dogs of various breeds were ultimately included in this study. Means with their standard errors for age and BW of the dogs were 6·2 (0·6) years (range 1·0–13·0 years) and 22·3 (2·5) kg (range 5·0–43·2 kg), respectively.

### Diet and feeding

Owners were provided a commercial dry extruded diet (Pedigree Complete Junior, chicken and rice; Masterfoods Veghel BV, The Netherlands) supplemented with TiO_2_ containing kibbles. The ingredients composition of the diet was: cereals (including 4 % rice), meat and animal derivatives (of which 4 % chicken), oils and fats (including 0·25 % fish oil and 0·2 % sunflower oil), vegetable products (of which 4·5 % dried beet pulp), minerals and vegetable protein extracts. Dogs were fed the diet for seven consecutive days at 480 kJ × BW^0·75^. Owners were instructed to carefully collect and store any leftovers. On day 7, owners were requested to collect all faeces during 24 h and store faeces at −20°C.

### Chemical analyses and calculations

The faeces were freeze-dried and ground over a 1 mm sieve in a Retsch mill (ZM100, Retsch B.V., Ochten, The Netherlands). The diet and faeces were analysed for DM, crude ash, N, crude fat (CF), crude fibre and Ti. DM and ash contents were determined by drying to a constant weight at 103°C and combustion at 550°C, respectively. Percentage of organic matter was calculated as: 100 − moisture% − ash%. Crude protein (6·25 N) was determined using the Kjeldahl method^(^^4^^)^ and CF was analysed according to the Berntop method^(^^5^^)^ with faecal samples being pre-digested with HCl. Crude fibre was analysed according to ISO 6865, 2000^(^^6^^)^. Ti was analysed using a modified method based on Short *et al*.^(^^7^^)^ and Myers *et al.*^(^^8^^)^. GE (kJ/g) was calculated using the equation (23·8 g crude protein) + (39·3 g CF) + (17·2(g nitrogen-free extract + g crude fibre))^(^^9^^)^. Apparent faecal digestibility (in percentage) was calculated as described by Bosch *et al.*^(^^10^^)^.

### Statistical analyses

All data were statistically analysed using SPSS 20·0 (IBM Software) software package. Descriptive statistics of the data were performed using the descriptive statistics procedure in SPSS. Regression analysis was performed using the linear regression procedure in SPSS. The level of significance was set at *P*<0·05. Plots were compiled using Microsoft Excel (Version 2010 for Windows).

## Results

The analysed composition of the diet was 33·7 % crude protein, 17·2 % CF, 2·0 % crude fibre and 7·3 % crude ash on a DM basis. The final dietary Ti content was 0·77 g/kg DM. The DM content was 92·8 % and the calculated GE content was 22·0 MJ/kg DM.

Data of thirty-nine dogs were used, because of erroneous analyses of the faecal sample from one of the dogs. Digestibility results of this study are shown in Table 1. Apparent digestibility of DM, GE, organic matter, N and CF showed relatively little variation (means with their standard errors 77·4 (0·79), 83·7 (0·71), 83·0 (0·61), 77·7 (0·81) and 94·3 (0·51), respectively). Analyses of crude fibre showed a wide variation (sem (4·85)), with an overall digestibility of 30·3 %.

**Table 1.** Means with their standard errors for apparent faecal digestibility percentage of a commercial canine diet fed to thirty-nine privately owned dogs

	Apparent digestibility	
Parameter	Mean	SEM
Gross energy	83·7	0·71
DM	77·4	0·79
Organic matter	83·0	0·61
Nitrogen	77·7	0·81
Crude fat	94·3	0·51
Crude fibre	30·3	4·85

**Fig. 1.**
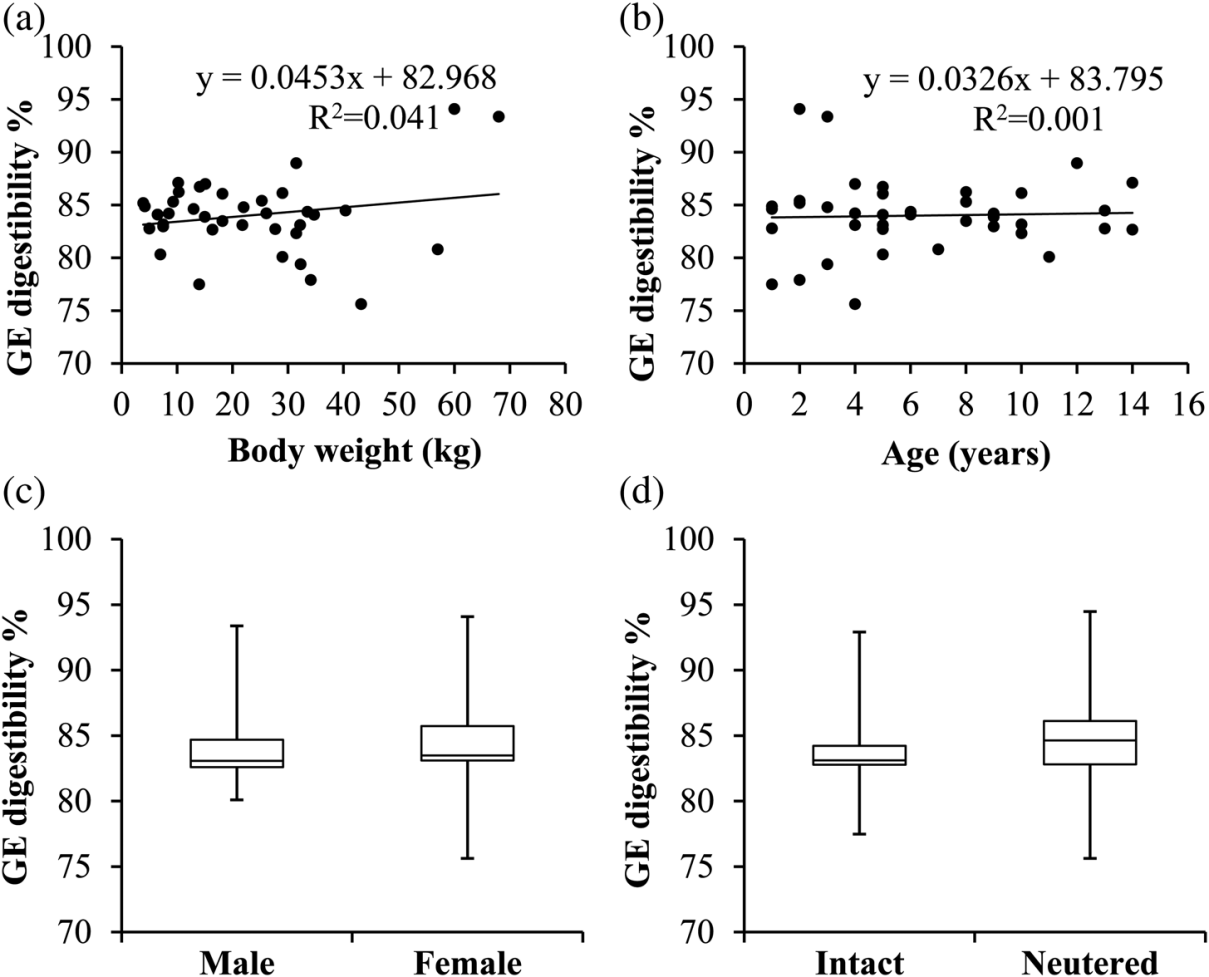
Apparent gross energy (GE) digestibility percentage of a commercial dry canine diet fed to thirty-nine privately owned dogs in relation to body weight (a), age (b), sex (c) and neuter status (d). In plots (c) and (d), the box represents the lower quartile, median and upper quartile, the whiskers extend to the minimal and maximal values. No statistical significant differences were found.

The correlation between BW and apparent faecal GE digestibility was low and not significant (*R*^2^ 0·041, Fig. 1(a)). For age, correlation with GE digestibility was also non-significant (*R*^2^ 0·001, Fig. 1(b)). Sex and neuter status did not significantly influence GE digestibility (Fig. 1(c) and (d)). Apparent digestibility of other nutrients was also not significantly influenced by weight, age, neuter status and sex.

## Discussion

The aim of this study was to report a simple and practical method for the analysis of apparent faecal digestibility of extruded pet food with TiO_2_ as an indigestible marker, which may be useful to evaluate differences in digestibility between dog breeds or dogs with various life stages. The effect of BW, age, sex and neuter status of the privately owned dogs on GE digestibility was studied to evaluate whether the effects reported under controlled conditions could be confirmed using this method with privately owned dogs. Digestibility of GE was not significantly affected by weight, age, sex or neuter status. The absence of an age effect observed in this study is in agreement with the current literature. A study by Taylor *et al.*^(^^11^^)^ did not observe significant differences in apparent faecal energy digestibility between young (<6 years, *n* 14) and older (>8 years, *n* 14) dogs of various breeds. A recent study by Zanatta *et al.*^(^^12^^)^ demonstrated that apparent faecal metabolisable energy digestibility did not differ between 6-month and 5-year-old Beagle dogs. In the same study, however, a significant higher CF digestibility was observed in puppies (95·3 %) compared with adult dogs (89·5 %), which is not in agreement with our data. The crude protein fraction in the study by Zanatta *et al.*^(^^12^^)^ showed a trend (*P* = 0·06) towards an increased digestibility with age, which is in agreement with the findings of our study.

The absence of a BW effect observed in our study is not in agreement with the study of Weber *et al.*^(^^2^^)^. In this study, it was demonstrated that apparent faecal GE digestibility was significantly higher in larger breed dogs compared with smaller breeds and significantly correlated with BW (*r* 0·66). This difference may be explained by the fact that the study of Weber *et al*. was conducted under controlled conditions and with growing puppies (up to 60 weeks of age) instead of mature dogs. Also, the limitation of 24-h faecal collection could explain the absence of BW effect in the data reported here. Meyer *et al.*^(^^3^^)^, in a study with ten canine breeds with BW ranging from 4·2 to 52·5 kg, reported no significant differences in apparent faecal digestibilities of the organic nutrients and GE among different breeds, despite large differences in BW between these breeds. This is in agreement with the data presented here. Further studies are required to elucidate the effect of BW on nutrient and GE digestibility.

To the authors’ knowledge, no data are available in the literature reporting the effect of sex and neuter status on faecal apparent GE digestibility in dogs. In growing pigs, however, Noblet *et al.*^(^^13^^)^ reported intact females having a significantly (*P* < 0·01) higher apparent faecal energy digestibility (86·3 %) compared with intact males (85·0 %). In the same study, no differences in apparent faecal energy digestibility were observed between intact and neutered male pigs (85·0 *v.* 85·1 %, respectively). The sex effect on energy digestibility observed by Noblet *et al*.^(^^13^^)^ is not in agreement with the results observed in our study.

The use of TiO_2_ as an inert marker has been validated in multiple species and is considered appropriate for use in faecal apparent digestibility studies^(^^14^^–^^16^^)^. The question remains whether an adjustment period of 7 d and 24 h faecal collection, which were used here, is sufficient to cover time variations in faecal nutrient and TiO_2_ excretion. In an early study by Lloyd & McCay^(^^17^^)^ in which the use of chromic oxide as an inert marker in dogs was validated, it was shown that a 3-d adjustment period was sufficient to obtain evenness of the distribution of this marker in the faeces. This would imply that a 7-d adjustment period, which was used in our study, may be more than sufficient. In the same study, it was shown that a collection period of 4 d was needed to compensate for day-to-day variations in chromic oxide and nutrient excretions. This may indicate that the 24-h collection period, which was used for our method, is not sufficient to compensate for variations in faecal output. To what extent this significantly affects the reliability of the method needs to be further studied.

It needs to be noted that the study design conducted here has several limitations. First of all, the data observed in this study were not validated with a standardised apparent faecal digestibility assay, using kennelled dogs in a laboratory setting. This may reduce the reliability of the data found in this study. Second, the 24-h collection period in this study may not be long enough to compensate for day-to-day variations in faecal output.

However, the digestibility assay using a practical approach described here may be a promising tool to determine digestibility of nutrients in dogs under free-living conditions rather than confined conditions of a laboratory setting. Careful instruction of participating dog owners is a prerequisite for compliance and reliability of the data. Also, a minimal collection period of 3 d instead of 24 h may be advisable to compensate for day-to-day variations in faecal output. Further studies need to compare this digestibility method to the standardised apparent faecal digestibility assay using total faecal collection.
